# Use of immuno-dominant epitope derived from genotype 4 as a diagnostic reagent for detecting the antibodies against Hepatitis E Virus

**DOI:** 10.1186/1743-422X-10-131

**Published:** 2013-04-25

**Authors:** Xiu Bing-shui, Feng Xiao-yan, He Jing, Chen Kun, Liu Jing, Dai Zhen-hua, Yang Xi-Qin, Wang Guo-hua, Wang You-chun, Zhang He-qiu, Song Xiao-guo, Zhu Cui-xia

**Affiliations:** 1 Institute of Basic Medical Sciences, Academy of military Medical Sciences, Taiping Road, Beijing, 100850, P.R. China; 2 Department of Cell Biology, National Institute for the Control of Pharmaceutical and Biological Products, No. 2 Tiantanxili, Beijing, 100050, P.R. China; 3 China National Center For Biotechnology Development, No. 6 West Fourth Ring Road, Beijing, 100036, P.R. China

**Keywords:** HEV, ORF2, ORF3, Genotype, Immunoassay

## Abstract

**Background:**

Despite the genotype 4 has become the dominant cause of hepatitis E disease in China, none antigen derived from genotype 4 of hepatitis E virus (HEV) was used in current commercial anti-HEV immunoassay, and the serological reactivity of antigen derive from genotype 4 is not well-charactered.

**Methods:**

We expressed and purified the 4 main immuno-dominant epitopes derived from genotype 1 and 4 including ORF2 (410-621aa) of genotype 4, ORF3 (47-114aa) of genotype 4, ORF2 (396-606aa) of genotype 1 and ORF3 (56-123aa) of genotype 4.

**Results:**

The ORF2 of genotype 4 displayed good diagnostics performance according to ROC analysis using in-house panel, and the immunoassays based the ORF2 of genotype 4 was then developed to detect the anti-HEV IgG antibodies and evaluated further in 530 anti-HEV IgG positive specimens and 380 negative specimens. The sensitivity and the specificity is 98.1% (520/530) and 94.7% (360/380) for immunoassay based on ORF2 of genotype 4, 96.6% (512/530) and 92.6% (352/380) for commercial immunoassay based on genotype 1. It is noted that all of the positive samples will be detected by combing two assays together. The anti-HEV immunoassays based on genotype 4 are in accordance with Chinese anti-HEV national standard,and show an good agreement of 95.8% with commercial assay (kappa=0.913, P=0.014).

**Conclusions:**

The immunoassay based on ORF2G4 displays good performance, and combining assay based on genotype 1 together with genotype 4 will benefit the HEV diagnosis in large scale samples.

## Background

Hepatitis E caused by Hepatitis E Virus (HEV) has been reported all over the world. Usually hepatitis E is endemic in developing countries associated with contaminated drinking water. In China, there are about 120,000 people infected with HEV and lead to 707 deaths in Xinjiang, during 1986–1988 [1]. In developed countries, hepatitis E occurs sporadically either related to travel to endemic areas or caused by autochthonous strains [2]. Now many animal including wild boars, deer, pig, horses, rabbits etc., was found to carry the virus, which is the potential reason contributed to the transmission of HEV [3,4].

The completion of the HEV genome facilitated the development of the HEV diagnostics. The HEV genome is a single-stranded, positive-sense RNA encoding three open reading frames (ORFs) named ORF1, ORF2 and ORF3 [5]. Now recombinant ORF2 and ORF3 antigens or immunodominant peptides were widely used in commercial HEV serological test including detecting IgM, IgG, IgA antibodies against HEV [6,7]. Recently, RT-PCR is a new way to detect HEV-RNA [8]. However, in addition to high expense and laborious work of the RT-PCR, HEV RNA exists only shortly in the blood and feces among sub-clinical cases [9]. Therefore, HEV immunoassays remain important and irreplaceable in the diagnosis of HEV infection especially in developing countries where HEV infection is often endemic.

Now four distinct genotypes (genotypes1-4) have been identified according to the phylogenic analyze of the HEV [10]. In spite only one serotype was found, recent report revealed that there are different antigenicity of HEV ORF2 between genotype 1 and 3 [11]. It is reported that anti-HEV were not detectable in a patient infected with HEV strain US-1 using an assay based on Burmese and Mexican strains [12]. Anti-ORF3 antibodies were detected in monkey infected with genotype 1 and 2 but not in monkey infected with genotype 3 or 4 [13]. All above evidences mean the sensitivity of the HEV serological test in definite geographic area is depend on the prevailed genotype and the immunodominant antigen used in the immunoassays.

Genotype 4 is originally identified in China in 2002, and with one nucleotide insertion in ORF2 which leaded to increased 13 amino acids at its C terminal comparing with other genotype [14]. Our collaborated research revealed that the ORF3 polypeptide of genotype 4 displayed stronger reactivity than that of genotype 1 in the sera from monkeys infected with genotype 4 [15]. Immunoassays based on ORF2 immuno-dominant epitope derived from HEV genotype 4 detected some cases of acute hepatitis E undetected by a commercial assay [16]. That means the antigen derived from genotype 4 is important in diagnosis anti-HEV especially in China where the genotype 4 and 1 were prevalent in recent report [17,18]. But until now, no commercial assay is developed based on antigen derived from genotype 4, and little is known about the sensitivity and specificity of immunoassay based on antigen derived from genotype 4 in large random samples of patients infected with HEV.

The aim of this study is to develop the immunoassay based on recombinant immuno-dominant HEV antigen derived from genotype 4 which has never been used in commercial test. Total of 910 samples were used to evaluate the sensitivity and specificity of the new HEV immunoassay comparing with commercial immunoassay based on genotype 1.

## Results

1. Selection and expression of HEV immuno-dominant epitopes derive from genotype 4 and 1.

**Figure 1:**
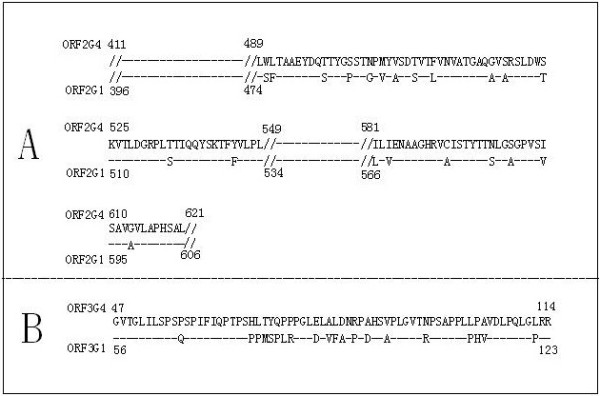
**The alignment of the deduced sequences of the amino acids of HEV sequences. A** is the alignment results of ORF2 between genotype 1 and 4; **B** is the alignment results of ORF3 between genotype 1 and 4; identical amino acids indicated by dashes.

**Figure 2:**
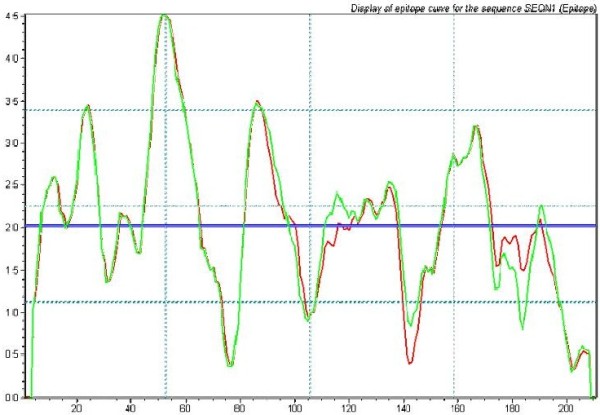
**The merge of hydrophobicity of ORF2 from genotype 1 and 4 analyzed by the BioSun software.** The green line is the hydrophobicity of ORF2 of genotype 1, and the red line is of genotype 4.

The selection of the immuno-dominant antigen is the key for the development of the immunoassay. The ORF2G1 (396-606aa, genotype 1) is well-characterized as an immunoreactive antigen and widely used in commercial anti-HEV immunoassay. Because the full length ORF2 from genotype 4 is 14 amino acids longer than that from genotype 1 for a single nucleotide insertion, the corresponding ORF2 immuno-domain of genotype 4 (ORF2G4) is 410-621aa. The sequences alignment revealed that there were 20 amino acids (9.52%) different between genotype 4 and 1 (Figure 1A), which lead to a little change in hydrophobicity analyzed by the BioSun software (Figure 2).

**Figure 3:**
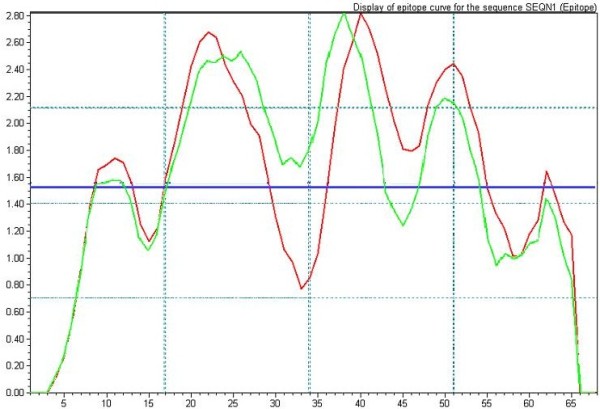
**The merge of hydrophobicity of ORF3 from genotype 1 and 4 analyzed by the BioSun software.** The green line is the hydrophobicity of ORF3 from genotype 1, and the red line is from genotype 4.

The nucleotide insertion of genotype 4 also changes the sequences of ORF3. The ORF3 of genotype 4 is 9 amino acids shorter than that from genotype 1 at the N terminal. However, polypeptide at the N terminal of ORF3 display no immunoreactive with anti- HEV positive sera including both genotype 4 (1-60aa) and genotype 1(1-51aa) in our collaborated researches [19]. The ORF3 (56-123aa) of genotype 1 (ORF3G1) and ORF3 (47-114aa) of genotype 4 (ORF3G4) were selected instead of the whole ORF3. The sequences alignment (Figure 1B) revealed that 20aa (29.4%) is different between he genotype 4 and 1 ORF3 selected, which lead to much change in hydrophobicity (Figure 3).

Above 4 antigens were expressed in insoluble form. Following purification, each product showed one clear band of the expected molecular mass on SDS–PAGE.

2. Reactivity of ORF2G1, ORF2G4, ORF3G1 and ORF3G4 detected by in-house panel.

**Figure 4:**
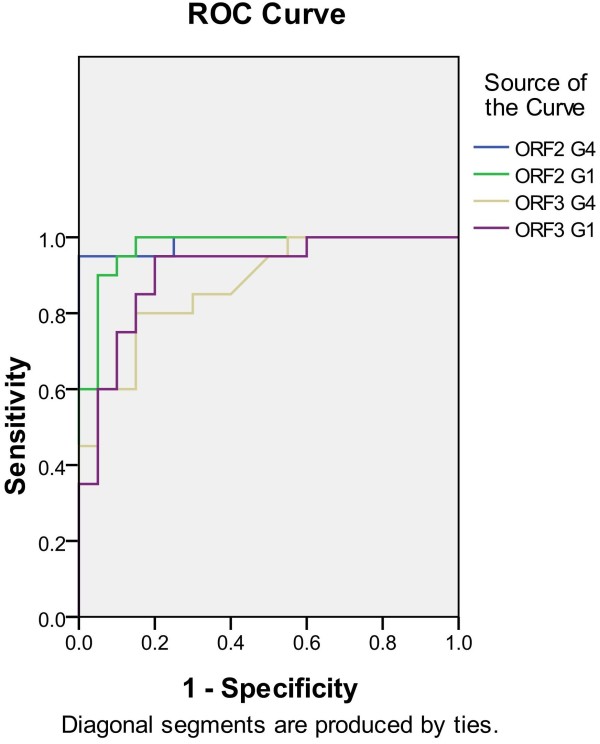
The ROC curve of the ORF2G1, ORF2G4, ORF3G1, ORF4G4 analyzed by SPSS software derived from an in-house panel.

The in-house panel was used to determine the reactivity of HEV by indirect ELISA including 20 anti-HEV IgG positive sera and 20 anti-HEV IgG negative sera. Both ORF2 antigens derived from genotype 1 and 4 display strong reactions with all of 20 anti-HEV positive sera. Comparably, the antigenicity of ORF3 is weaker than that of ORF2. Only 12 of 20 positive sera were reacted with ORF3. There are no sera positive with ORF3 and negative with ORF2. That means that adding ORF3 antigen could have little help to acquire high sensitivity of anti-HEV detection in the in-house panel. However, there are 2 HEV positive sera display inconsistent results between the ORF3 antigen of genotype 1 and 4 (Table 1). Further ROC curve showed that the ORF2G4 achieved best performance with area under the curve 0.988 (Figure 4), and was selected to develop the HEV immunoassay.

3. Establish and evaluate HEV–IgG Immunoassay based on the ORF2G4 antigen.

**Table 1:** Comparison between the ORF3G1 and ORF3G4 by ELISA in in-house panel

**ORF3G1**	**ORF3G4**	
	**Positive**	**Negative**
**Positive**	11	1
**Negative**	1	22

400 plasma of health blood donor were used to determine the cut-off value of the assay. The cut-off value was 0.18 based on the average value plus triple standard deviation according to the statistical analysis. The specimen with S/CO ratio≥1.0 is positive, the specimen with S/CO ratio< 1.0 is negative.

The Chinese National Reference was used to evaluate the performance of our immunoassay based on genotype 4. There are 30 true negative, 10 positive specimens, 4 sera with serial dilution, 1 precision specimens in Chinese National Reference for anti-HEV antibody detection. Our result is as follows: all of the positive and negative specimens were correctly identified. The sensitivity test using the sera with serial dilution is 1:16 and precision CV is 4%. These results are consistent with the national standard.

We use a total of 910 specimens to evaluate our anti- HEV immunoassay. 530 HEV IgG positive samples and 380 negative samples (190 HBV infected sample, 190 health blood donor) were included. The results were shown in Table 2. The sensitivity of IgG assay based on genotype 4 is 98.1% (520/530) in the anti-IgG positive sample, which is little higher than that of commercial immunoassay based on genotype 1 (96.6%, 512/530)(*χ*^2^=2.384, P=0.125). The specificity of assay based on genotype 4 and 1 is 94.7% (360/380) and 92.6% (352/380) respectively in anti-IgG negative panel. There are no significant difference between two assays(*χ*^2^=1.767, P=0.184).

**Table 2:** The results of anti-HEV IgG Immunoassay based on genotype 1 and 4

**Group**	**Number**	**positive by assay based on genotype 1**	**positive by assay based on genotype 4**
HEV IgG positive	530	512	520
HBV	190	15	9
blood donor	190	13	11

To better understand the influence brought by different HEV genotype antigen, we analyzed the agreement between the 2 immunoassay in positive and negative samples respectively. The results were summarized in Table 3. The two tests gave an excellent agreement of 95.8% with a kappa statistic of 0.913 (P=0.014) in 910 samples. It is noted there is no samples negative with both test in positive panel and the detectable rates could reach 100% in 530 positive samples when combining two assays together.

**Table 3:** Agreement between immunoassay based on HEV genotype 1 and 4

**Immunoassay based on genotype 4**	**Immunoassay based on genotype 1**	
	**+**	**-**
+	502+*19*	18+*1*
-	10+*9*	0+*351*

The normal number is the value derive from 530 HEV positive samples, and the italic and underline number is the value derive from 380 HEV negative samples.

## Discussion

The antigen used in the immunoassay is the key to acquire high sensitivity and specificity. It was reported that ORF2 (394-606aa) of the genotype 1 expressed in E.coli contained the conformational neutralizing epitopes, and display high immuno-reactivity with both acute and convalescent sera of HEV patients [20]. But the full-length ORF2 expressed will mask some epitope, and display weak immunoreactivity in detecting anti-HEV antibodies [21]. Now, ORF2 (394-606aa) of the genotype 1 is the main antigen widely used in the diagnosis of anti-HEV antibodies [22]. However, the valuable epitopes derived from genotype 4 has not yet been well investigated. Using the conformational antibodies 1G10 of genotype 4, ORF2 (477-613aa) was confirmed as conformational-dependent epitope, and its flanking amino acid residues is important to form the stable conformation of the epitope [23]. Our previous research found the ORF2 (384-673aa) of genotype 4 predicted by bio-information comprise epitope mentioned above and displayed good reactivity with serum of infected patients and monkey inoculated with HEV experimentally. In this paper, we confirmed the results again that the ORF2 (410-621aa) of genotype 4 is immunoreactive just as ORF2 (374-606aa) of genotype 1.

It is doubtful about the diagnostics value of the ORF 3 antigen used in anti-HEV detection. Some authors reveal that the assay based on ORF3 could detect some early antibodies negative with ORF2 [24,25]. While others found the kits using both ORF3 and ORF2 did not display more sensitive than the one used ORF2 alone [26]. As samples used were from convalescent and not early infected patients, we found there were no samples with anti-ORF3 positive but anti-ORF2 negative results in this study. However, our data revealed that the ORF3 of genotype 1 and 4 displayed different reactive patterns in the in-house panel, and it is reported that the ORF3 display genotypic difference in others’ researches [19]. That suggested the ORF3 antigen could be of potential use in HEV serotype detection.

Anti-IgG was mainly appear in the convalescent serum,while anti-HEV IgG also can be detected in some acute case patient [27]. That means the HEV IgG detection was used not only in epidemiology of HEV but also in diagnosis of acute HEV infection. Although there is no statistical significance, the anti-HEV assays based on genotype 4 detected little more positive samples than commercial assay did. Interestingly, some samples failed to detect in commercial assay based on genotype 1 could be detectable by the assay based on genotype 4, and the sensitivity would be improved to 100% if we combined two assays together. There are two reason contributed for the adding effect. One is the different antigenicity of HEV ORF2 between different genotype. According to the epidemiological study,the genotype 1 and 4 are prevalent in China. That means using of antigen derived from genotype 1 and 4 will benefit the HEV detection in China. It is reported that anti-HEV were not detectable in a patient infected with HEV genotype 3 using an assay based on other genotype [12]. Therefore, it is necessary to develop the HEV immunoassay based on four different genotype discovered. The other reason is the various technology and procedure used by two test lead to adding affect of the diagnosis. It is well-known that the WHO recommends using 2 ELISA tests to reduce the HCV transmission by blood compounds [28]. Now, it is urged by some researchers that HEV will be transmitted by blood especially in epidemic area [29]. Our study provided the new HEV immunoassay which could complement for current commercial test.

## Conclusion

In this research, we evaluated the main immuodominant epitope of HEV ORF2 and ORF3 derived respectively from genotype 4 and genotype 1. The immunoassay based on ORF2 of genotype4 (410-621aa) was developed and displayed good performance comparing with commercial kits based on genotype1 in 910 clinical samples and blood donor. Our results reveal that combing assay based on genotype 1 together with the one based on genotype 4 will benefit the HEV diagnosis especially in large scale samples.

## Materials and methods

### Specimens

A total of 910 specimens were used to evaluate the anti-HEV immunoassay: 530 anti-HEV IgG positive specimens were from convalescent patients, 190 negative specimens were collected from healthy blood donors and 190 from HBV patients. The specimens were provided by Institute of Hepatitis, Youan Hospital, Beijing, China; Anhui Province Municipal Hospital, Hefei, China; and Shanghai public health center, Shanghai, China. 400 specimens of healthy blood donor were used for calculating the cut-off value provided by the Blood Center of Beijing Red Cross. 20 samples with anti-HEV IgG antibody positive and 20 negative samples were used as lab panel to screening the epitopes were provided by Youan Hospital. These blood and plasma specimens were stored at −70°C. This study was approved by the Ethics Committees of the related hospitals and research institutes.

### HEV antigen preparation

Total of 4 antigens were selected in this study including ORF2 derived from genotype 1 [GenBank: M73218] (396-606aa, ORF2G1), ORF2 derived from genotype 4 [GenBank: AJ272108] (410-620aa, ORF2G4), ORF3 derived from genotype 1 [GenBank: M73218] (1-123aa, ORF3G1), ORF3 derived from genotype 4 [GenBank: AJ272108] (47-114aa, ORF3G4). The above 4 gene fragments were PCR from HEV genotype 4 or genotype 1 T-easy vector, and inserted into the pBVIL-1 vector. All of the 4 antigens were expressed as fusion polypeptides with an additional 17.5 kDa of interleukin (IL)-1βand were purified successfully using ionexchange chromatography and were then freeze-dried.

### Indirect ELISA to detect the HEV- IgG

Microplates coating with 0.25 μg recombinant HEV antigen were incubated in 100 mmol/L phosphate buffer (pH7.4) overnight at 4°C, and blocked with 2% BSA in the phosphate buffer at 4°C for 3h. 100 μL of the sample were added at 1:10 dilution in the buffer (100 mmol/L sodium phosphate buffer pH7.5 containing 10% goat serum and 0.05% Tween) at 37°C for 1h. the 100 mmol/L sodium phosphate buffer (pH7.5) containing 0.05% Tween were used to wash the plates five times. After incubated for 30 min at 37°C with 1:25000 diluted HRP-conjugated monoclonal antibodies against human IgG, the plates were washing again. After the visualized reaction with substrate buffer (50 mmol/L sodium phosphate-citric acid buffer, pH 5.0 containing 0.4 mg/mL TMB and 0.4 μL/mL of 30% hydrogen peroxide), the reaction was stopped by 50 μL of 2 mol/L sulfuric acid, and the OD450 was measured in a microplate ELISA reader. The cut-off value was calculated according to method 2.4.

### The evaluation of the immunoassay with the China National anti-HEV Panel

The China national anti-HEV panel was used to assess anti-IgG HEV immunoassays based on ORF2G4. The panel included 30 true negative, 10 positive specimens, 1 specimens with dilution at 1:4, 1:8, 1:16, 1:32, and 1 precision specimens repetitively tested by 10 time. The standards of the China National anti-HEV IgG Panel were as follows: negative ratio is equal or more than 29/30, and the positive ratio is 10/10; the sensitivity test is more than 1/16 and precision CV is lower than 15% (n = 10).

### Comparison of the immunoassay with other commercial HEV ELISA kits

The anti-HEV IgG antibody detection kit (indirect assay, Wan Tai Pharmaceutical Co., Lot number: EG 20090704, Xiamen, China) based on ORF2 of genotype 1 was used to evaluated the HEV antibodies assay based on HEV ORF2 of genotype 4. The above assays were performed according to the manufacturer’s instructions.

### Statistics

All data were analyzed by statistical software SPSS 16.0. Receiver operator characteristic curve (ROC) was conducted to evaluate the diagnostic significance of recAg-1-12. Kappa test was conducted to evaluate the consistency of qualitative results. P<0.05 was considered statistically significant.

## Abbreviations

HEV: Hepatitis E virus; ORF: The open reading frame; ORF2G4: The ORF2 of genotype 4; ORF3G4: The ORF2 of genotype 4; ORF2G1: The ORF2 of genotype 1; ORF3G1: The ORF3 of genotype 1.

## Competing interests

The authors declare that they have no competing interest.

## Authors’ contributions

XB-s and FX-y carried out the molecular genetic and bioinformatics studies. HJ and CK carried out the immunoassays. LJ and DZ-h participated in the sequence alignment. YX-c and WG-h purified the recombinant antigen. SX-g, ZC-x carried the SAS-PAGE. WY-c and ZH-q participated in the design of the study and coordination and helped to draft the manuscript. All authors read and approved the final manuscript.

## References

[B1] Aye TT Uchida T Ma XZ Lida F Shikata T Zhuang H Win KM Complete nucleotide sequence of a hepatitis E virus isolated from the Xinjiang epidemic (1986–1988) of China Nucleic Acids Res 1992 20 3512 3512 10.1093/nar/20.13.3512 1630924 PMC312512

[B2] Aggarwal R Naik S Epidemiology of hepatitis E: current status J Gastroenterol Hepatol 2009 24 9 1484 1493 10.1111/j.1440-1746.2009.05933.x 19686410

[B3] Geng J Wang L Wang X Fu H Bu Q Liu P Zhu Y Wang M Sui Y Zhuang H Potential risk of zoonotic transmission from young swine to human: seroepidemiological and genetic characterization of hepatitis E virus in human and various animals in Beijing. China J Viral Hepat 2011 18 10 e583 e590 10.1111/j.1365-2893.2011.01472.x 21914080

[B4] Geng J Wang L Wang X Fu H Bu Q Zhu Y Zhuang H Study on prevalence and genotype of hepatitis E virus isolated from Rex Rabbits in Beijing, China J Viral Hepat 2011 18 9 661 667 10.1111/j.1365-2893.2010.01341.x 20609076

[B5] Reyes GR Huang CC Yarbough PO Tam AW Hepatitis E virus. Comparison of 'New and Old World' isolates J Hepatol 1991 13 Suppl 4 S155 S161 1822510 10.1016/0168-8278(91)90050-l

[B6] Takahashi M Kusakai S Mizuo H Suzuki K Fujimura K Masuko K Sugai Y Aikawa T Nishizawa T Okamoto H Simultaneous detection of immunoglobulin A (IgA) and IgM antibodies against hepatitis E virus (HEV) Is highly specific for diagnosis of acute HEV infection J Clin Microbiol 2005 43 1 49 56 10.1128/JCM.43.1.49-56.2005 15634950 PMC540162

[B7] Zhang S Tian D Zhang Z Xiong J Yuan Q Ge S Zhang J Xia N Clinical significance of anti-HEV IgA in diagnosis of acute genotype 4 hepatitis E virus infection negative for anti-HEV IgM Dig Dis Sci 2009 54 11 2512 2518 10.1007/s10620-008-0657-4 19117132

[B8] Wu KT Chung KM Feng IC Sheu MJ Kuo HT Koay LB Lin CY Tang LY Tsai SL Acute hepatitis E virus infection in Taiwan 2002–2006 revisited: PCR shows frequent co-infection with multiple hepatitis viruses J Med Virol 2009 81 10 1734 1742 10.1002/jmv.21442 19697413

[B9] Aggarwal R Kini D Sofat S Naik SR Krawczynski K Duration of viraemia and faecal viral excretion in acute hepatitis E Lancet 2000 356 9235 1081 1082 10.1016/S0140-6736(00)02737-9 11009149

[B10] Purcell RH Emerson SU Hepatitis E: an emerging awareness of an old disease J Hepatol 2008 48 3 494 503 10.1016/j.jhep.2007.12.008 18192058

[B11] Schlauder GG Mushahwar IK Genetic heterogeneity of hepatitis E virus J Med Virol 2001 65 2 282 292 10.1002/jmv.2031 11536234

[B12] Jiménez De Oya N Galindo I Gironés O Duizer E Escribano JM Saiz JC Serological immunoassay for detection of hepatitis E virus on the basis of genotype 3 open reading frame 2 recombinant proteins produced in Trichoplusia ni larvae J Clin Microbiol 2009 47 10 3276 3282 10.1128/JCM.00750-09 19656986 PMC2756903

[B13] Zhou YH Purcell RH Emerson SU A truncated ORF2 protein contains the most immunogenic site on ORF2: antibody responses to non-vaccine sequences following challenge of vaccinated and non-vaccinated macaques with hepatitis E virus Vaccine 2005 23 24 3157 3165 10.1016/j.vaccine.2004.12.020 15837215

[B14] Wang Y Zhang H Ling R Li H Harrison TJ The complete sequence of hepatitis E virus genotype 4 reveals an alternative strategy for translation of open reading frames 2 and 3 J Gen Virol 2000 81 Pt 7 1675 1686 10859372 10.1099/0022-1317-81-7-1675

[B15] Ma H Song X Harrison TJ Zhang H Huang W Wang Y Hepatitis E virus ORF3 antigens derived from genotype 1 and 4 viruses are detected with varying efficiencies by an anti-HEV enzyme immunoassay J Med Virol 2011 83 5 827 832 10.1002/jmv.22032 21360543

[B16] Wang Y Zhang H Li Z Gu W Lan H Hao W Ling R Li H Harrison TJ Detection of sporadic cases of hepatitis E virus (HEV) infection in China using immunoassays based on recombinant open reading frame 2 and 3 polypeptides from HEV genotype 4 J Clin Microbiol 2001 39 12 4370 4379 10.1128/JCM.39.12.4370-4379.2001 11724847 PMC88551

[B17] Dong C Dai X Shao JS Hu K Meng JH Identification of genetic diversity of hepatitis E virus (HEV) and determination of the seroprevalence of HEV in eastern China Arch Virol 2007 152 4 739 746 10.1007/s00705-006-0882-0 17131064

[B18] Lam WY Chan RC Sung JJ Chan PK Genotype distribution and sequence variation of hepatitis E virus, Hong Kong Emerg Infect Dis 2009 15 5 792 794 10.3201/eid1505.081579 19402972 PMC2687042

[B19] Ma H Song X Li Z Harrison TJ Zhang H Huang W Hao W Kong W Wang Y Varying abilities of recombinant polypeptides from different regions of hepatitis E virus ORF2 and ORF3 to detect anti-HEV immunoglobulin M J Med Virol 2009 81 6 1052 1061 10.1002/jmv.21484 19382255

[B20] Li F Zhuang H Kolivas S Locarnini SA Anderson DA Persistent and transient antibody responses to hepatitis E virus detected by western immunoblot using open reading frame 2 and 3 and glutathione S-transferase fusion proteins J Clin Microbiol 1994 32 9 2060 2066 7529246 10.1128/jcm.32.9.2060-2066.1994 PMC263942

[B21] Li F Torresi J Locarnini SA Zhuang H Zhu W Guo X Anderson DA Amino-terminal epitopes are exposed when full-length open reading frame 2 of hepatitis E virus is expressed in Escherichia coli, but carboxy-terminal epitopes are masked J Med Virol 1997 52 3 289 300 10.1002/(SICI)1096-9071(199707)52:3<289::AID-JMV10>3.0.CO;2-E 9210039

[B22] Hu WP Lu Y Precioso NA Chen HY Howard T Anderson D Guan M Double-antigen enzyme-linked immunosorbent assay for detection of hepatitis E virus-specific antibodies in human or swine sera Clin Vaccine Immunol 2008 15 8 1151 1157 10.1128/CVI.00186-07 18495846 PMC2519296

[B23] Zhang H Dai X Shan X Meng J The Leu477 and Leu613 of ORF2-encoded protein are critical in forming neutralization antigenic epitope of hepatitis E virus genotype 4 Cell Mol Immunol 2008 5 6 447 456 10.1038/cmi.2008.56 19118511 PMC4072421

[B24] Bendall R Ellis V Ijaz S Ali R Dalton H A comparison of two commercially available anti-HEV IgG kits and a re-evaluation of anti-HEV IgG seroprevalence data in developed countries J Med Virol 2010 82 5 799 805 10.1002/jmv.21656 20336757

[B25] Zhang JZ Im SW Lau SH Chau TN Lai ST Ng SP Peiris M Tse C Ng TK Ng MH Occurrence of hepatitis E virus IgM, low avidity IgG serum antibodies, and viremia in sporadic cases of non-A, -B, and -C acute hepatitis J Med Virol 2002 66 1 40 48 10.1002/jmv.2109 11748657

[B26] Ghabrah TM Tsarev S Yarbough PO Emerson SU Strickland GT Purcell RH Comparison of tests for antibody to hepatitis E virus J Med Virol 1998 55 2 134 137 10.1002/(SICI)1096-9071(199806)55:2<134::AID-JMV9>3.0.CO;2-3 9598934

[B27] Huang S Zhang X Jiang H Yan Q Ai X Wang Y Cai J Jiang L Wu T Wang Z Guan L Shih JW Ng MH Zhu F Zhang J Xia N Profile of acute infectious markers in sporadic hepatitis E PLoS One 2010 5 10 e13560 10.1371/journal.pone.0013560 21042408 PMC2958841

[B28] Safe Blood Donation Safe Blood and Blood products: Identifying low risk donors 2002 WHO Module 2: Anonymous pp. 83 pp. 84

[B29] Khuroo MS Kamili S Yattoo GN Hepatitis E virus infection may be transmitted through blood transfusions in an endemic area J Gastroenterol Hepatol 2004 19 7 778 784 10.1111/j.1440-1746.2004.03437.x 15209625

